# A Platina Case

**Published:** 1885-12

**Authors:** 


### A PLATINA CASE.

Dr. Seutin, of Brussels (*Revue Hom. Belge*, July, 1885, p. 113), in an article on Platina relates the following case:

A lady suffered for several days from an intense uterine hæmorrhage. The apparently most appropriate remedies, as Aconite, Belladonna, Ipecacuanha, Pulsatilla, Sabina, Secale, and finally Cinchona, produced only transitory amelioration, always followed by renewal of the severe flooding. Seutin felt puzzled about his failure, and in a fresh examination the patient indulged in extravagant praise of her own family—her husband, her children, her brothers and sisters. This tendency to vanity and boasting led the Doctor to prescribe Platina, sixth, two drops in one hundred and eighty grammes distilled water, a tablespoonful every hour for a few hours and then in lengthened intervals. After twelve hours all flooding had stopped, she felt well, and only for sake of precaution she was advised to keep quiet for a week.

He agrees with Hahnemann, Hering, and Hughes that Platina is to females what Aurum is to the male sex, and in chronic ovarian affections Platina holds the same place which Aurum occupies in corresponding affections of the testicles.

The old school has spoken lately in high terms of Platina chloride in chronic syphilis and condylomata. In a score of obstinate cases, which failed to yield to Potassium iodide, a prompt cure followed the use of Iodide and Chloride of Platina, five drops of the third decimal dilution twice a day. Teste, in his *Systematisation pratique*, places Platina next to Thuja as antisycotic.

